# Hotel Dieu

**Published:** 1830-05

**Authors:** 


### HOTEL DIEU.

### *Internal Strangulation, with Gangrene and Laceration of the Ileum. General Peritonitis.**

Although internal strangulations of the intestines usually bid defiance to all the resources of our art, such diseases are highly interesting in a medico-legal point of view. They frequently occur very suddenly, in the midst of the most perfect health; the patient expires in dreadful torment, vomiting until his last breath, and, in fact, presenting all the phenomena which are observed in cases of poisoning. The records of medicine contain many cases of this kind, but it is still desirable to add to the number, as from their comparative study may possibly result more satisfactory conclusions than have yet been derived.

Antoine Bernard, ætat. fifty-seven, having usually enjoyed a tolerable state of health, states that he was perfectly well on Wednesday, January 6th, when he was attacked very suddenly in the evening with violent colic, which was quickly accompanied by vomiting and repeated evacuations from the bowels. He thought his illness arose from indigestion, and was satisfied with taking some mild and warm fluid. The vomiting increased during the night, and continued without intermission for several days: no other remedies being applied but rest, strict diet, and simple ptisans. On the 9th, he was admitted into the Hôtel Dieu.

His face was shrunk, skin cold, thready and quick pulse; belly excessively painful, the weight of the bedclothes being almost insupportable; abdomen also rather tense. Bowels not open since the first day of the attack; the vomiting was almost incessant, the matter thrown up consisted of mucus mingled with feces. The moment the patient ventured to appease the distressing thirst with which he was tormented, nausea and vomiting occurred. The abdomen was covered with warm fomentations, the extremities were kept warm, and a few spoonsful of syrup of white poppies were given. Clysters with laudanum were also administered, but were not retained.

In the evening of the following day, there was a general warmth diffused over the surface of the body, and the patient felt better. Still, however, the countenance was expressive of great anxiety; there was a sharpness about the nose, and the forehead was covered with a clammy sweat. He hiccuped incessantly, and the

* Laucette Française.

exhaustion that was felt from the constant nausea led the patient to fear that his malady was incurable.

The above remedies were continued, but without advantage. The general weakness hourly increased, and on the 13th the patient was relieved from his sufferings by death. The sudden accession of symptoms which evidently depended upon mischief in the digestive organs could only be attributed to two circumstances: the introduction of poison, or a mechanical impediment to the passage of the fecal matter. There was no reason to suspect that poison had been taken, either from any thing that the patient had said, or from the existence of any special symptoms indicative of the fact. Every probability was in favor of the second supposition, and the dissection proved that it was well founded.

The body was examined about twelve hours after death. Head and chest healthy. The abdomen, although depressed, was rather sonorous on percussion. The mouth was full of yellow matter, similar to that which had been vomited during life. The peritoneum lining the abdominal muscles adhered in various parts to the intestines. In the cavity of the abdomen there were collections of pus, and the pelvis was filled with dark-looking matter, much resembling thick ink. The stomach was small in size; its mucous membrane patched with dark brown spots; the rugæ near the pyloric portion very red. The duodenum, and first half of the small intestines, were filled with yellow "bouillie," like that which the patient had vomited, the quantity of it increasing towards the large intestines. There was also some air in the small intestines, which accounted for the tympanitic appearance that had been observed. About four feet from the ileo-cæcal valve, the intestine was obstructed by an apparently fibrous band, which completely surrounded it, and held it fixed towards the vertebræ. This band was not more than fifteen or eighteen lines long, and one or two broad; it resembled a nerve both in feel and appearance; it passed from one point of the mesentery to another, forming a kind of arch, under which was contained the strangulated portion of intestine. At a short distance, the same intestine was grasped by another and similar band. At least three fourths of the caliber of the gut was closed by this accidental ligature, and thus the fecal matter had been interrupted in its passage. The interval between the two bands was about ten inches. Proceeding from the first, the caliber of the intestine was found to be much diminished, and the mucous membrane of a livid colour. About five inches lower than the first band was found a large irregular opening in the gut, with ragged, black, and fetid edges. The succeeding portion of intestine was contracted like that above, to the next point of strangulation, where the second preternatural band was found. The large intestines were somewhat contracted, and contained a small quantity of dry, well-formed feces. The other viscera healthy. The whole of the intestines were of a deep black colour. There were numerous adhesions between the different reflections of the peritoneum, and between these adhesions were several small abscesses. The peritoneum was remarkably thin, and tore from the slightest touch.

This case shows the importance of carefully investigating the cause of death, even when no therapeutic benefit is likely to reward our trouble. In the present day no surgeon would think of opening the abdomen to destroy the mechanical cause of strangulation;* but we may learn to distinguish these spontaneous diseases from the effects of poison, which the public are always quick to suspect when sudden symptoms are rapidly followed by death. A short time ago, Mademoiselle Hullin, of the French Opera, died under precisely similar circumstances to those related in the above case, and her death gave rise to the most unfounded suspicions, which were only allayed by the testimony of Orfila and Rostan. In this case there was an adventitious band which had strangulated the ileum, and which had alone given rise to the symptoms which had been confidently attributed to poison, not only by the friends of the patient, but by the physicians who were first consulted.†

* Vide Stephens on Obstructed and Inflamed Hernia, and on Mechanical Obstructions of the Bowels internally, p. 125 *et seq.* Mr. S. recommends that an opening should be made into the abdomen in such cases. We confess we doubt the propriety of such an operation, but the remarks made by Mr. Stephens are worth attention.—Editor.

† Dr. Christison refers to this case in his Treatise on Poisons, p. 101. He states his belief that stercoraceous vomiting is never caused by poisoning, and, as this symptom occurred in the case of Mademoiselle H., Dr. C. thinks it might have been held sufficient to settle the real nature of it.—Editor.

